# Willingness to pay for medications among patients with Rome IV Irritable Bowel Syndrome

**DOI:** 10.1111/nmo.14483

**Published:** 2022-09-30

**Authors:** Vivek C. Goodoory, Cho Ee Ng, Christopher J. Black, Alexander C. Ford

**Affiliations:** ^1^ Leeds Institute of Medical Research at St. James's University of Leeds Leeds UK; ^2^ Leeds Gastroenterology Institute St. James's University Hospital Leeds UK; ^3^ County Durham and Darlington NHS Foundation Trust Durham UK

**Keywords:** abdominal pain, constipation, diarrhea, irritable bowel syndrome, medication, willingness to pay

## Abstract

**Background:**

Little is known about willingness to pay for medications among individuals with irritable bowel syndrome (IBS).

**Methods:**

We collected demographic, gastrointestinal symptom, psychological health, quality of life, and healthcare usage data from 752 adults with Rome IV‐defined IBS. We examined willingness to pay for a hypothetical medication in return for improvement in IBS symptoms using a contingent valuation method, according to these variables.

**Results:**

The median amount of money individuals was willing to pay was £1–£50 (IQR £0–£100) per month for a medication with a 100% chance of improving IBS symptoms. Women, compared with men, (92.7% willing to pay “£0,” 89.8% “£1–£50,” 87.3% “£51–£100,” 78.9% “£101–£200,” and 78.5% “more than £200,” *p* = 0.008) were less likely to be willing to pay for a pill with a 100% chance of improving IBS symptoms whilst those with an annual income of £30,000 or more (12.2% willing to pay “£0,” 25.2% “£1–£50,” 33.5% “£51–£100,” 40.2% “£101–£200,” and 35.1% “more than £200,” *p* = 0.002) were more likely. We observed a higher willingness to pay among those with lower IBS‐related quality of life (*p* = 0.002 for trend). Of all 752 individuals, 92.7%, 74.5%, and 58.0% would be willing to pay for a medication that would give them a 100%, 50%, or 30% chance of improving IBS symptoms, respectively.

**Conclusion:**

Patients with IBS are willing to pay for medications which improve IBS symptoms. Future studies should investigate the relative importance of medication pricing, efficacy, and side effect profile among individuals with IBS.

## Graphical abstract

This cross‐sectional study recruited 752 individuals with Rome IV‐defined IBS to examine their willingness to pay for medications that improve the symptoms of IBS and to examine individuals’ preference for medications.

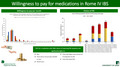

**Abbreviations**
FODMAP: fermentable oligosaccharides, disaccharides, monosaccharides, and polyols
GP: general practitioner
HADS: hospital anxiety and depression scale
IBS: irritable bowel syndrome
IBS‐C: irritable bowel syndrome with constipation
IBS‐D: irritable bowel syndrome with diarrhea
IBS‐M: irritable bowel syndrome with mixed bowel habits
IBS‐QOL: irritable bowel syndrome quality of life
IBS‐SSS: irritable bowel syndrome severity scoring system
IQR: interquartile range
NHS: National Health Service
NICE: National Institute for Health and Care Excellence
PHQ‐12: patient health questionnaire‐12
SD: standard deviation
VSI: visceral sensitivity index

**Key Points**

- There is limited knowledge about the willingness to pay for medications in IBS. We conducted a cross‐sectional study among individuals with Rome IV‐defined IBS to examine their willingness to pay for a hypothetical medication.
- Participants were willing to pay a median £1‐£50 per month for a hypothetical medication with a 100% chance of improving IBS symptoms. We observed several factors associated with a higher willingness to pay.
- These results have important implications for pharmaceutical companies and regulatory agencies.

## 1 INTRODUCTION

Irritable bowel syndrome (IBS) is a disorder of gut‐brain interaction characterized by recurrent abdominal pain in association with abnormal stool form or frequency.^1^ It affects between 5% and 10% of the world's population.^2^, ^3^, ^4^ A diagnosis of IBS is reached using symptom‐based criteria proposed by the Rome Foundation, the Rome IV criteria,^5^ in patients with typical symptoms in the absence of red flags and limited investigations.^6^, ^7^ IBS is a chronic disorder with a relapsing and remitting course,^8^ partly because of the modest efficacy of medications.^9^, ^10^, ^11^, ^12^, ^13^ Current treatment strategies aim to alleviate the predominant gastrointestinal symptom(s) reported by patients.^14^, ^15^ Patients exhibit reductions in quality of life of the same magnitude as those with organic gastrointestinal disorders, such as Crohn's disease.^16^ IBS affects people in their activities of daily living and at work,^17^, ^18^ and costs an estimated £1.3– £2 billion per year to the UK National Health Service (NHS).^19^ In an attempt to improve their health, individuals with IBS are willing to accept substantial risks from medications.^20^, ^21^, ^22^

In the UK, the Medicines and Healthcare products Regulatory Agency is the responsible agency to ensure medicines meet an acceptable standard of safety and efficacy.^23^ In addition, the National Institute for Health and Care Excellence (NICE), considers the cost of these medications to decide whether they should be funded by the NHS using public funds.^24^ Partly as a result of cost, patients' access to some drugs is limited because they can only be initiated in secondary care, rather than by general practitioners (GPs). This has led some pharmaceutical companies to cease promotion of certain drugs for IBS or even, in the case of lubiprostone and eluxadoline, withdraw them from the market. Even though lay persons and patients are involved in decisions taken by NICE, there are a lack of data on willingness to pay to guide an acceptable threshold for cost that can be used to determine access to medications in the NHS. Additionally, because several medications for IBS are bought by patients over the counter, it is crucial to evaluate individual's willingness to pay from their own pocket for drugs.

A previous cross‐sectional study reported that among individuals with IBS whose annual salary was <$75,000 (approximately £62,000), the willingness to pay for a medication to improve IBS symptoms was between $49.4 (approximately £41) and $73.3 (approximately £61) per month depending on symptoms.^22^ However, the study findings were limited by the relatively small population used. In addition, the authors did not examine willingness to pay according to other demographic characteristics, IBS characteristics, or psychological factors that are commonly associated with IBS. We, therefore, examined these issues in a cross‐sectional study recruiting a large cohort of individuals with IBS. We hypothesized that most individuals with IBS would be willing to pay for a hypothetical medication with a 100% chance of improving IBS symptoms, but that this may be affected by personal finances, symptom severity, psychological health, and IBS‐related quality of life.

## 2 METHODS

### 2.1 Participants and Setting

We recruited individuals registered with ContactME‐IBS, a national UK registry run by County Durham and Darlington NHS Foundation Trust, whose members have IBS and are interested in research.^25^ We have reported data from this cohort previously.^17^, ^19^, ^20^ Briefly, the registry advertises itself to individuals in the community via numerous sources including posters in primary care, specialist hospital clinics, pharmacies, or social media. Individuals enroll by completing a short questionnaire about bowel symptoms and providing contact details. Among all 4280 registrants, 2268 (53%) have seen their GP with IBS, and another 1455 (34%) a gastroenterologist. There were no exclusion criteria for this study apart from the inability to understand written English. We contacted all registered individuals, via electronic mailshot, in July 2021, directing them to an online questionnaire and information about the study. All responses were stored in an online database and non‐responders received a reminder email in August 2021. Participants were given a chance to win one of three gift cards (worth £200, £100, or £50) in return for completing the questionnaire. The University of Leeds research ethics committee approved the study in March 2021 (MREC 20–051).

### 2.2 Data Collection and Synthesis

#### 2.2.1 Demographic and Symptom Data

We collected basic demographic data, including age, sex, lifestyle (tobacco and alcohol consumption), ethnicity, marital status, educational level, and annual income. We defined presence of IBS according to the Rome IV questionnaires,^26^ assigning the presence or absence of Rome IV‐defined IBS among all individuals according to the scoring algorithm proposed for its use.^5^ We categorized IBS subtype according to the criteria recommended in the questionnaire, using the proportion of time stools looked abnormal according to the Bristol stool form scale. We asked all participants to choose their most troublesome symptom from a list of five possibilities, including abdominal pain, constipation, diarrhea, bloating, or urgency.

IBS Symptom Severity, Mood and Somatic Symptoms, Gastrointestinal Symptom‐specific Anxiety, and IBS‐ related Quality of Life.

We used validated questionnaires, as we have described previously.^17^, ^19^, ^20^ Briefly, we assessed severity of symptoms using the IBS severity scoring system (IBS‐SSS),^27^ which carries a maximum score of 500 points, with <75 points indicating remission; 75–174 points mild; 175–299 points moderate; and 300–500 points severe symptoms. We used the hospital anxiety and depression scale (HADS) to collect anxiety and depression data. The total HADS score ranges from 0 to 21 for either anxiety or depression. We categorized severity for each into normal (total HADS depression or anxiety score 0–7), borderline normal,^8^, ^9^, ^10^ or abnormal (≥11).^28^ We collected somatic symptom‐reporting data using the patient health questionnaire‐12 (PHQ‐12),^29^ derived from the validated PHQ‐15.^30^ The total PHQ‐12 score ranges from 0 to 24. We categorized severity into high (total PHQ‐12 ≥ 13), medium,^8^, ^9^, ^10^, ^11^, ^12^ low,^4^, ^5^, ^6^, ^7^ or minimal (≤3). We used the visceral sensitivity index (VSI),^31^ which measures gastrointestinal symptom‐specific anxiety. We divided these data into equally sized tertiles, as there are no validated cut offs to define low, medium, or high levels of gastrointestinal symptom‐specific anxiety. Finally, we used the irritable bowel syndrome quality of life (IBS‐QOL) to measure health‐related quality of life,^32^, ^33^ with a total possible score of 0–136 and lower scores indicating better quality of life. Scores were transformed to a 0–100‐point scale with zero indicating worst quality of life and 100 indicating best quality of life. We divided these data into equally sized tertiles, as there are no validated cut offs to define low, medium, or high levels of quality of life.

#### 2.2.2 IBS‐related Resource Use

We collected data on healthcare usage related to a person's IBS over the 12 months prior to recruitment. We asked participants to report any appointments with healthcare professionals (GPs, gastroenterologists, specialist nurses, dietitians, or psychologists), including the number of appointments, number of investigations (blood tests, stool tests, endoscopies, abdominal ultrasounds, computed tomography scans, magnetic resonance imaging scans, hydrogen breath tests, or 23‐seleno‐25‐homo‐tauro‐cholic acid scans), number of unplanned emergency department attendances or inpatient admissions (including length of stay), and over the counter and prescribed medication usage (in months). We applied costs for GP appointments from Unit Costs of Health and Social Care 2020,^34^ and other appointments, investigations, or unplanned inpatient days in secondary care using the NHS's 2019/20 National Cost Collection Data.^35^ We assumed all appointments for IBS were follow‐up appointments, which cost less than a new patient appointment. We applied the lowest price for a 1‐month supply of each IBS‐related medication using the online version of the British National Formulary.^36^

#### 2.2.3 Willingness to Pay for Improvement of IBS Symptoms

We used a contingent valuation method. This is a technique in which respondents are asked to state their preferences in a hypothetical scenario, to determine the amount of their own money they were willing to pay per month for a hypothetical medication in return for a 100% chance of improving IBS symptoms. As there is no validated questionnaire to assess IBS patient's willingness to pay for medication, we used a set of questions to examine participants' willingness to pay with potential responses on a 12‐point scale from “not willing to pay anything” to “more than £500” with each response in between representing equal £50 increments (e.g., “£1–£50,” “£51–100,” “£101–£150”). We also examined how much money participants were willing to pay per month if the medication only had a 30% or 50% chance of improving IBS symptoms.

#### 2.2.4 Choice of pill

We asked participants to choose a pill they would prefer to take from a list of eight pills. Four pills relieved one symptom (pain, bloating, diarrhea, or constipation) almost completely, but hardly relieved other symptoms, whilst the other four pills relieved one symptom (pain, bloating, diarrhea, or constipation) well and relieved other symptoms a little.

### 2.3 Statistical Analysis

Because data were skewed positively, we categorized individuals in groups of those who were willing to pay “£0,” “£1–£50,” “£51–£100,” “£101–£200,” and “more than £200.” We examined characteristics of participants in each of these groups. We also examined the pill participants would prefer according to IBS subtype and most troublesome symptom. We compared categorical variables such as sex, ethnicity, IBS subtype, IBS‐SSS severity, presence or absence of abnormal anxiety or depression scores, levels of somatic symptom‐reporting, levels of gastrointestinal symptom‐specific anxiety, and levels of quality of life using a χ^2^ test and continuous data such as age, mean annual cost of medications for IBS, and mean annual direct healthcare cost of IBS using one‐way analysis of variance for continuous data. Statistical significance was defined as a *p* value <0.01.

## 3 RESULTS

In total, 1278 (29.9%) of 4280 registrants completed the questionnaire. Of these, 752 (58.8%) met Rome IV criteria for IBS (mean age 45.3 years (range 18–81 years), 655 (87.1%) female). In total, 136 (18.1%) had IBS with constipation (IBS‐C), 306 (40.7%) IBS with diarrhea (IBS‐D), and 301 (40.0%) IBS with mixed bowel habits (IBS‐M). The median annual income of respondents was £20,000–£29,999 (interquartile range [IQR] £10,000–£39,999). The median amount of their own money individuals with IBS was willing to pay per month for a hypothetical medication with a 100% chance of improving their symptoms was £1–£50 (IQR £0–£100).

### 3.1 Willingness to Pay for a Hypothetical Medication with a 100% Chance of Improving IBS Symptoms

We examined the characteristics of the 752 individuals with Rome IV IBS according to the amount of money they were willing to pay per month for a hypothetical medication with a 100% chance of improving IBS symptoms (Table 1). Women (92.7% willing to pay “£0,” 89.8% “£1–£50,” 87.3% “£51–£100,” 78.9% “£101–£200,” and 78.5% “more than £200,” *p* = 0.008) were significantly less likely to be willing to pay for a pill with a 100% chance of improving IBS symptoms than men. Alcohol users (30.9% willing to pay “£0,” 58.5% “£1–£50,” 64.7% “£51–£100,” 67.4% “£101–£200,” and 50.8% “more than £200,” *p* < 0.001) and individuals with annual income of £30,000 or more (12.2% willing to pay “£0,” 25.2% “£1–£50,” 33.5% “£51–£100,” 40.2% “£101–£200,” and 35.1% “more than £200,” *p* = 0.002) were willing to pay significantly more for a medication with a 100% chance of improving IBS symptoms. Willingness to pay for a 100% chance of improvement of IBS symptoms was not associated with age, marital status, level of education, IBS subtype, most troublesome symptom, duration of IBS, or number of drugs taken for IBS in the last 12 months. Individuals who were willing to pay more for a hypothetical medication with a 100% chance of improving IBS symptoms had significantly higher mean annual costs for IBS medications (mean £82.67 (standard deviation [SD] £136.63) for those willing to pay “£0,” £61.34 (SD 66.84) for “£1–£50,” £76.74 (SD 101.84) for “£51–£100,” £64.74 (SD £68.32) for “£101–£200,” and £127.82 (SD £170.09) for “more than £200,” *p* < 0.001) and generally higher mean direct healthcare costs for IBS, although the latter was not statistically significant (*p* = 0.06). There were significantly higher proportions of individuals with severe IBS (*p* < 0.001 for trend) and higher somatic symptom‐reporting scores (*p* = 0.004 for trend) among individuals willing to pay “£0” or “more than £200” compared with those willing to pay “£1–£50,” “£51–£100,” or “£101–£200.” We observed a similar trend among those with higher depression scores, but this did not reach statistical significance (*p* = 0.01). Finally, there was a significantly higher proportion of those willing to pay more for a medication with a 100% chance of improving IBS symptoms with lower IBS‐related quality of life (*p* = 0.002 for trend).

**TABLE 1:** Characteristics of individuals with Rome IV IBS according willingness to pay per month for a hypothetical medication with a 100% chance of improving symptoms

	Willingness to pay per month for a medication with a 100% chance of improving symptoms					*p* Value
	£0 (*n* = 55)	£1–£50 (*n* = 364)	£51–£100 (*n* = 173)	£101–£200 (*n* = 95)	More than £200 (*n* = 65)	
Female (%)	51 (92.7)	327 (89.8)	151 (87.3)	75 (78.9)	51 (78.5)	0.008
Mean age (SD)	50.3 (15.5)	45.2 (14.4)	44.9 (14.4)	44.5 (15.8)	44.3 (14.3)	0.52
White ethnicity (%)	53 (96.4)	354 (97.3)	169 (97.7)	94 (98.9)	59 (90.8)	0.04
Married (%)	32 (58.2)	241 (66.2)	114 (65.9)	62 (65.3)	38 (58.5)	0.62
Smoker (%)	8 (14.5)	31 (8.5)	24 (13.9)	10 (10.5)	9 (13.8)	0.28
Alcohol user (%)	17 (30.9)	213 (58.5)	112 (64.7)	64 (67.4)	33 (50.8)	<0.001
University or postgraduate level of education (%)	19 (34.5)	142 (39.0)	79 (45.7)	45 (47.4)	29 (44.6)	0.30
Annual income of £30,000 or more (%)	6 (12.2)	83 (25.2)	53 (33.5)	35 (40.2)	20 (35.1)	0.002
IBS subtype at baseline (%)						
Constipation	7 (13.5)	65 (17.9)	29 (17.0)	23 (24.5)	12 (19.0)	0.32
Diarrhea	21 (40.4)	140 (38.6)	72 (42.1)	42 (44.7)	31 (49.2)	
Mixed stool pattern	24 (46.2)	158 (43.5)	70 (40.9)	29 (30.9)	20 (31.7)	
Most troublesome symptom (%)						
Abdominal pain	14 (25.5)	68 (18.7)	47 (27.2)	21 (22.1)	19 (29.2)	0.53
Constipation	3 (5.5)	25 (6.9)	13 (7.5)	6 (6.3)	6 (9.2)	
Diarrhea	9 (16.4)	50 (13.7)	29 (16.8)	20 (21.1)	9 (13.8)	
Bloating/distension	14 (25.5)	117 (32.1)	47 (27.2)	26 (27.4)	14 (21.5)	
Urgency	15 (27.3)	104 (28.6)	37 (21.4)	22 (23.2)	17 (26.2)	
Duration of IBS diagnosis, year(s) (%)						
<5 years	10 (18.2)	70 (19.2)	39 (22.5)	19 (20.0)	15 (23.1)	0.87
5 years or more	45 (81.8)	294 (80.8)	134 (77.5)	76 (80.0)	50 (76.9)	
Number of drugs in the last 12 months (%)						
0	9 (16.4)	47 (12.9)	25 (14.5)	11 (11.6)	4 (6.2)	0.18
1	11 (20.0)	110 (30.2)	33 (19.1)	24 (25.3)	11 (16.9)	
2	14 (25.5)	87 (23.9)	47 (27.2)	29 (30.5)	19 (29.2)	
3	9 (16.4)	54 (14.8)	34 (19.7)	17 (17.9)	15 (23.1)	
4	3 (5.5)	35 (9.6)	22 (12.7)	7 (7.4)	9 (13.8)	
5 or more	9 (16.4)	31 (8.5)	12 (6.9)	7 (7.4)	7 (10.8)	
Mean annual cost of medications for IBS, SD (£UK)	82.67 (136.63)	61.34 (66.84)	76.74(101.84)	64.74 (68.32)	127.82 (170.09)	<0.001
Mean annual direct healthcare cost of IBS, SD (£UK)	577.39 (761.54)	489.39 (947.85)	533.83 (1093.15)	611.96 (1184.41)	895.59 (1138.78)	0.06
IBS‐SSS severity at baseline (%)						
Mild	3 (5.6)	46 (12.7)	27 (15.8)	5 (5.3)	5 (7.8)	<0.001
Moderate	14 (25.9)	160 (44.2)	67 (39.2)	44 (46.8)	15 (23.4)	
Severe	37 (68.5)	156 (43.1)	77 (45.0)	45 (47.9)	44 (68.8)	
HADS‐A categories at baseline (%)						
Normal	9 (16.4)	102 (28.0)	52 (30.1)	21 (22.1)	16 (24.6)	0.54
Borderline	17 (30.9)	80 (22.0)	41 (23.7)	21 (22.1)	15 (23.1)	
Abnormal	29 (52.7)	182 (50.0)	80 (46.2)	53 (55.8)	34 (52.3)	
HADS‐D categories at baseline (%)						
Normal	21 (38.2)	208 (57.1)	100 (57.8)	47 (49.5)	28 (43.1)	0.010
Borderline	13 (23.6)	68 (18.7)	40 (23.1)	29 (30.5)	15 (23.1)	
Abnormal	21 (38.2)	88 (24.2)	33 (19.1)	19 (20.0)	22 (33.8)	
PHQ‐12 severity at baseline (%)						
Low	2 (3.6)	14 (3.8)	9 (5.2)	10 (10.5)	1 (1.5)	0.004
Mild	9 (16.4)	83 (22.8)	38 (22.0)	31 (32.6)	15 (23.1)	
Moderate	22 (40.0)	157 (43.1)	79 (45.7)	31 (32.6)	18 (27.7)	
Severe	22 (40.0)	110 (30.2)	47 (27.2)	23 (24.2)	31 (47.7)	
VSI at baseline (%)						
Low	16 (29.1)	140 (38.5)	54 (31.2)	22 (23.2)	15 (23.1)	0.03
Medium	20 (36.4)	113 (31.0)	63 (36.4)	32 (33.7)	19 (29.2)	
High	19 (34.5)	111 (30.5)	56 (32.4)	41 (43.2)	31 (47.7)	
IBS‐QOL (%)						
Low	20 (36.4)	102 (28.0)	50 (28.9)	32 (33.7)	35 (53.8)	0.002
Medium	16 (29.1)	127 (34.9)	53 (30.6)	37 (38.9)	19 (29.2)	
High	19 (34.5)	135 (37.1)	70 (40.5)	26 (27.4)	11 (16.9)	

* *p* Value for Pearson χ^2^ for comparison of categorical data and one‐way analysis of variance for continuous data.

### 3.2 Willingness to Pay for a Hypothetical Medication with Varying Chance of Improvement of IBS symptoms

**FIGURE 1:**
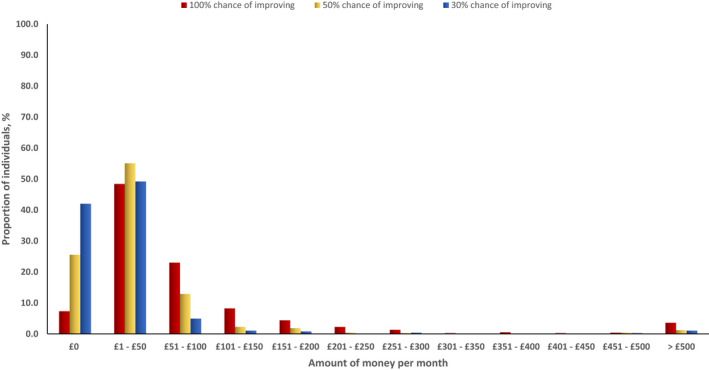
Amount of money per month individuals with Rome IV IBS are willing to pay for a hypothetical medication with a 100%, 50%, or 30% chance of improving symptoms of IBS

We examined the proportion of individuals who were willing to pay for a hypothetical medication with a 100%, 50%, or 30% chance of improving symptoms of IBS. The median amount of money individuals was willing to pay were £1–£50 (IQR £0–£50), 1‐£50 (IQR £0–£50), and 1‐£50 (IQR £0–£100) for a 30%, 50%, and 100% chance of improvement of IBS symptoms respectively. Figure 1 shows the proportion of individuals with Rome IV IBS according to the amount of money they would be willing to spend per month for each medication. Of 752 people, 55 (7.3%), 192 (25.5%), and 316 (42.0%) were not willing to pay anything for a medication that would give them a 100%, 50%, or 30% chance of improving IBS symptoms respectively. Conversely, 697 (92.7%), 560 (74.5%), and 436 (58.0%) individuals were willing to pay for a medication that would give them a 100%, 50%, or 30% chance of improving IBS symptoms, respectively. For a 100% chance of improvement of IBS symptoms, 21.3% of the 752 participants were willing to pay more than £100 per month and 13.0% were willing to pay £150 per month.

### 3.3 Choice of pill

**FIGURE 2:**
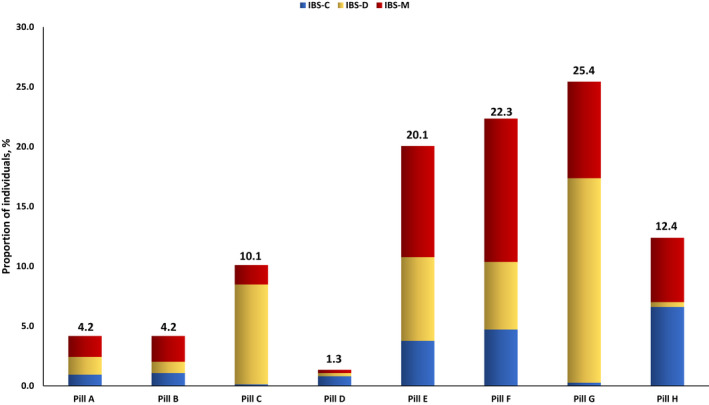
Choice of pill among individuals with Rome‐IV IBS ACCORDING to IBS subtype. Pill A**—**relieves pain almost completely; hardly relieves bloating, diarrhea, or constipation; Pill B**—**relieves bloating almost completely; hardly relieves pain, diarrhea, or constipation; Pill C**—**relieves diarrhea almost completely; hardly relieves pain, bloating, or constipation; Pill D**—**relieves constipation almost completely; hardly relieves pain, bloating, or diarrhea; Pill E**—**relieves pain well; relieves bloating, diarrhea, or constipation a little; Pill F—relieves bloating well; relieves pain, diarrhea, or constipation a little; Pill G—relieves diarrhea well; relieves pain, bloating, or constipation a little; Pill H—relieves constipation well; relieves pain, bloating, or diarrhea a little

**FIGURE 3:**
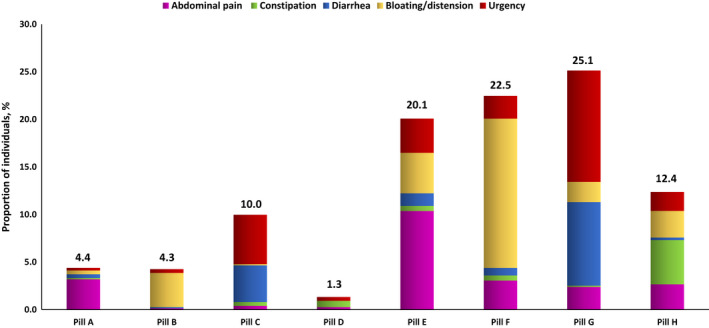
Choice of pill among individuals with Rome‐IV IBS according to most troublesome symptom. Pill A**—**relieves pain almost completely; hardly relieves bloating, diarrhea, or constipation; Pill B**—**relieves bloating almost completely; hardly relieves pain, diarrhea, or constipation; Pill C**—**relieves diarrhea almost completely; hardly relieves pain, bloating, or constipation; Pill D**—**relieves constipation almost completely; hardly relieves pain, bloating, or diarrhea; Pill E**—**relieves pain well; relieves bloating, diarrhea, or constipation a little; Pill F—relieves bloating well; relieves pain, diarrhea, or constipation a little; Pill G—relieves diarrhea well; relieves pain, bloating, or constipation a little; Pill H—relieves constipation well; relieves pain, bloating, or diarrhea a little

When asked about their preference out of eight pills, 602 (80.1%) individuals chose one of the four pills that relieved pain, bloating, diarrhea, or constipation well and relieved other symptoms a little (pills E to H), whereas 150 (19.9%) chose one of the other four pills that relieved pain, bloating, diarrhea, or constipation almost completely, but hardly relieved other symptoms (pills A to D) (Figure 2). We observed a significant difference in the choice of pill among individuals with different IBS subtypes (*p* < 0.001 for trend) (Table 2) and those who reported different symptoms as their most troublesome (*p* < 0.001 for trend) (Figure 3 and Table 3). For example, among the 306 individuals with IBS‐D, 189 (61.8%) chose either pill C or pill G, both of which relieved diarrhea primarily. However, 127 (67.2%) of these individuals preferred pill G, which relieved diarrhea well but also relieved other symptoms of IBS a little, whereas 62 (32.8%) chose pill C, which relieved diarrhea almost completely but hardly relieved other symptoms.

**TABLE 2:** Choice of pill among individuals with Rome IV IBS according to IBS subtype

	IBS subtype			*p* Value
	IBS‐C (*n* = 136)	IBS‐D (*n* = 306)	IBS‐M (*n* = 301)	
Pill A—relieves pain almost completely; hardly relieves bloating, diarrhea, or constipation.	7 (5.1)	11 (3.6)	13 (4.3)	<0.001
Pill B—relieves bloating almost completely; hardly relieves pain, diarrhea, or constipation.	8 (5.9)	7 (2.3)	16 (5.3)	
Pill C—relieves diarrhea almost completely; hardly relieves pain, bloating, or constipation.	1 (0.7)	62 (20.3)	12 (4.0)	
Pill D—relieves constipation almost completely; hardly relieves pain, bloating, or diarrhea.	6 (4.4)	2 (0.7)	2 (0.7)	
Pill E—relieves pain well; relieves bloating, diarrhea, or constipation a little.	28 (20.6)	52 (17.0)	69 (22.9)	
Pill F—relieves bloating well; relieves pain, diarrhea, or constipation a little.	35 (25.7)	42 (13.7)	89 (29.6)	
Pill G—relieves diarrhea well; relieves pain, bloating, or constipation a little.	2 (1.5)	127 (41.5)	60 (19.9)	
Pill H—relieves constipation well; relieves pain, bloating, or diarrhea a little.	49 (36.0)	3 (1.0)	40 (13.3)	

* *p* Value for Pearson χ^2^ for comparison of categorical data.

**TABLE 3:** Choice of pill among individuals with Rome IV IBS according to most troublesome symptom

	Most troublesome symptom					*p* Value
	Abdominal pain (*n* = 169)	Constipation (*n* = 53)	Diarrhea (*n* = 117)	Bloating/Distension (*n* = 218)	Urgency (*n* = 195)	
Pill A—relieves pain almost completely; hardly relieves bloating, diarrhea, or constipation.	24 (14.2)	1 (1.9)	3 (2.6)	3 (1.4)	2 (1.0)	<0.001
Pill B—relieves bloating almost completely; hardly relieves pain, diarrhea, or constipation.	1 (0.6)	0 (0.0)	1 (0.9)	27 (12.4)	3 (1.5)	
Pill C—relieves diarrhea almost completely; hardly relieves pain, bloating, or constipation.	3 (1.8)	3 (5.7)	29 (24.8)	1 (0.5)	39 (20.0)	
Pill D—relieves constipation almost completely; hardly relieves pain, bloating, or diarrhea.	2 (1.2)	5 (9.4)	0 (0.0)	0 (0.0)	3 (1.5)	
Pill E—relieves pain well; relieves bloating, diarrhea, or constipation a little.	78 (46.2)	4 (7.5)	10 (8.5)	32 (14.7)	27 (13.8)	
Pill F—relieves bloating well; relieves pain, diarrhea, or constipation a little.	23 (13.6)	4 (7.5)	6 (5.1)	118 (54.1)	18 (9.2)	
Pill G—relieves diarrhea well; relieves pain, bloating, or constipation a little.	18 (10.7)	1 (1.9)	66 (56.4)	16 (7.3)	88 (45.1)	
Pill H—relieves constipation well; relieves pain, bloating, or diarrhea a little.	20 (11.8)	35 (66.0)	2 (1.7)	21 (9.6)	15 (7.7)	

* *p* Value for Pearson χ^2^ for comparison of categorical data.

## 4 DISCUSSION

This cross‐sectional study recruited 752 individuals with Rome IV‐defined IBS to examine willingness to pay for a medication that improves the symptoms of IBS. The median amount of money individuals was willing to pay was £1–£50 per month for a medication with a 100% chance of improving IBS symptoms. Men, individuals earning £30,000 or more annually, those with higher mean costs for IBS medications in the last 12 months, and those with lower IBS‐related quality of life were willing to pay more for a medication with a 100% chance of improving IBS symptoms. There were significantly higher proportions of individuals with more severe IBS or higher somatic symptom‐reporting scores among those who were not willing to pay anything (“£0”) and those who were willing to pay the most (“more than £200”). The willingness of individuals to pay for medication decreased with decreasing likelihood of the pill's ability to relieve their symptoms, with over 40% of individuals not willing to pay anything for a medication that gave only a 30% chance of IBS symptoms improving. Finally, 80% of individuals preferred a pill that would relieve one symptom well but also improve others, reflecting the constellation of symptoms that make up IBS, and those with different IBS subtypes or predominant symptoms generally chose pills that would relieve symptoms they were most likely to experience or found the most troublesome.

This study recruited a large cohort of individuals who self‐identified as having IBS and also met Rome IV criteria. Our sample included participants from different age groups, levels of education, income brackets, and IBS severity and duration, suggesting a wide range of individuals have been included. Importantly, we included not only those who had seen a gastroenterologist or GP for IBS, but also individuals who had not consulted a doctor. We used validated questionnaires, which have been used previously in studies in IBS and other gastrointestinal disorders. We obtained near complete data for variables of interest because we used mandatory fields in our online questionnaire. Finally, we used a contingent valuation method to estimate willingness to pay, which has been used widely in health‐economic studies,^37^ making it clear in every question that the participants would have to purchase the hypothetical pill using money out of their own pocket.

There are some important limitations. Because we recruited individuals from a national UK registry of participants with IBS to reduce selection bias, we were unable to check medical records to rule out other organic diseases that present with similar symptoms, such as celiac disease or inflammatory bowel disease.^38^, ^39^ However, we believe this is unlikely to have affected our results. Firstly, IBS is more prevalent than these conditions. Secondly, UK national guidance recommends these conditions are ruled out prior to making a diagnosis of IBS.^14^, ^15^ Thirdly, almost 90% of the members of ContactME‐IBS have seen a GP or a gastroenterologist for IBS. Finally, over 80% of participants had a diagnosis or IBS for 5 years or more. As all participants were UK residents and nearly 97% were White, the results of our study should be interpreted with caution in other countries or ethnic groups. The possibility of a hypothetical bias cannot be ruled out, given no real payment was involved, and our participants may have overlooked budgetary constraints, thereby overestimating willingness to pay. Conversely, using an online questionnaire may have underestimated willingness to pay. One study demonstrated willingness to pay for healthcare services increased when participants were interviewed face‐to‐face, rather than completing an online questionnaire.^40^ As the NHS in the UK is free at the point of use and collects a fixed charge for NHS prescriptions from patients, participants in this study may be unfamiliar with costs of healthcare services and medications, and therefore the concept of willingness to pay. This means the results may not be generalizable to other countries or healthcare systems. Although we presented efficacy of the hypothetical medication to our participants, we did not incorporate the safety profile. Finally, we did not consider other treatment options available for IBS such as cognitive behavioral therapy or a diet low in fermentable oligosaccharides, disaccharides, monosaccharides, and polyols (FODMAPs).^11^, ^12^ These have other costs associated with them, including travel to, as well as missed work and child care costs for, appointments or higher costs for low FODMAP foods although, in particular, patients may prefer dietary therapies over drug treatments.^41^

To the best of our knowledge, only one previous study has examined willingness to pay for medications among individuals with IBS.^22^ This study recruited patients with Rome‐IV defined IBS from secondary care, examining willingness to pay for a hypothetical medication that would improve pain, bloating, diarrhea, or constipation stratified by annual income. Among individuals with an annual salary less than $75,000 (approximately £62,000), the willingness to pay for a medication to improve IBS symptoms was of a similar magnitude to our results varying between approximately £40 and £60 per month, depending on symptoms. Our study not only recruited a larger sample of individuals with Rome IV IBS, but also examined impact of baseline characteristics, IBS subtype, predominant symptom, severity and duration of IBS, IBS‐related quality of life, cost of participants' IBS‐related medications and direct healthcare cost of IBS in the last 12 months, and psychological co‐morbidities on willingness to pay.

Over 90% of individuals with IBS were willing to pay for a medication with a 100% chance of improving IBS symptoms, with 4% willing to pay >£500 per month for this medication. The fact some individuals with IBS were willing to give up >£500 worth of other activities in life to improve their symptoms demonstrates the substantial opportunity costs arising from the condition. In addition to previous studies highlighting the risk of death individuals with IBS are willing to accept in return for cure of symptoms,^20^, ^21^, ^22^ the current study highlights some individuals with IBS are willing to take substantial financial risks even without a 100% chance of symptom improvement. Those with higher annual income, or worse IBS‐related quality of life reported higher willingness to pay compared with their counterparts. This is probably because those with a worse quality of life are more likely to be willing to spend more to improve their health, and those with higher pay have more disposable income to spend on improving symptoms. Our observation that over 40% of individuals were not willing to pay anything for a medication that offered them only a 30% chance of symptom improvement is also important, given that most currently available drugs in IBS have limited efficacy and a similarly modest chance of improving symptoms.^9^, ^10^, ^11^, ^12^, ^13^ It is possible that individuals with IBS are not aware of the limited efficacy of current medications and, hence, are not willing to pay for medications that seem to offer only a small theoretical chance of improving symptoms. Additionally, individuals in the UK may not be willing to pay for medications, not necessarily because they cannot afford to do so, but because they are used to either a standard nominal fee for all medications or are eligible for free prescriptions.

Our results suggest there may be several factors involved in financial decision‐making to help improve symptoms of IBS including patient demographics, disease characteristics, psychological co‐morbidities, perceived benefits of medications, and attitude toward paying for healthcare. The fact there were higher proportions of individuals with more severe IBS or higher somatic symptom‐reporting scores among those not willing to pay anything (“£0”) and among those willing to pay the most (“more than £200”), with a similar trend among those with higher depression scores, is interesting. One possible explanation is that in those with more severe IBS, higher levels of somatic symptom‐reporting, or higher depression scores, there is one group of individuals who are willing to accept huge financial risks to improve their symptoms, demonstrating the lengths they are willing to go to improve their disease state, and another group who are not willing to spend any money at all, perhaps as a result of their disappointment with previous therapies they have tried for IBS, which were not efficacious. We did not observe the same trend among those with low IBS‐related quality of life, with only a significantly higher proportion of those with low IBS‐related quality of life in the group willing to pay “more than £200.” From this, we infer it is not necessarily severity of gastrointestinal or extraintestinal symptoms related to IBS that drives willingness to pay for medication, but their impact on quality of life. We have demonstrated previously that direct healthcare costs of IBS are higher among those with lower IBS‐related quality of life.^19^ Our observation in the present study that willingness to pay is associated with direct healthcare costs of IBS may be because IBS‐related quality of life is a confounding factor. Equally, it is possible that individuals who have utilized healthcare services more extensively have a better understanding of the costs involved, compared with those who have not. Lastly, our observation that most individuals preferred a pill that improved more than one symptom, even if none of the symptoms are completely relieved, accurately reflects the multi‐faceted symptom burden of IBS. This highlights the importance global assessment of symptoms and supports the use of composite endpoints in trials.

The results of this study have several important implications. Although the analysis of willingness to pay represents individual or societal preferences, it is likely to influence decisions made by agencies such as NICE, especially given that lay persons and patients are involved regarding funding of medications in the NHS. As patients are taking an increasingly active role in medical decision‐making, and because cost is an important factor in choosing a treatment option, clinicians should consider individuals' willingness to pay, especially in countries without free healthcare or patients who do not have health insurance. Individuals' willingness to pay is also an important consideration for pharmaceutical companies when setting the price of medications for IBS. This is especially important because of the high costs of over‐the‐counter medications. Given most medications for IBS have an efficacy rate of only around 30%, and that over 40% of individuals in our study were not willing to pay anything for a medication of that efficacy, pharmaceutical companies should consider recalibrating pricing of medications and advertise their efficacy data more clearly to individuals buying their products.

In summary, our results show that over 90% of individuals with Rome IV IBS were willing to pay for a hypothetical medication with a 100% chance of improving their symptoms. Men, those with higher annual income, those with higher mean cost of IBS medications used in the last 12 months, and those with lower IBS‐related quality of life were willing to pay significantly more for a medication with a 100% chance of improving IBS symptoms. Although the cost of medication is important, future studies should examine how much consideration individuals with IBS give to medication pricing compared with other factors, such as efficacy and side effect profile.

## AUTHOR CONTRIBUTIONS

VCG, CEN, CJB, and ACF conceived and drafted the study. VCG and CEN collected all data. VCG, CJB, and ACF analyzed and interpreted the data. VCG and ACF drafted the manuscript. All authors have approved the final draft of the manuscript. Potential competing interests: Vivek C. Goodoory: none. Cho Ee Ng: none. Christopher J. Black: none. Alexander C. Ford: none.

## FUNDING INFORMATION

Unrestricted research monies were provided by Tillotts Pharma UK Ltd. The funder had no input into the concept, design, analysis, or reporting of the study.

## CONFLICTS OF INTEREST

Guarantor of the article: ACF is guarantor. All author have no conflict of interest.
